# Generative model based on junction tree variational autoencoder for HOMO value prediction and molecular optimization

**DOI:** 10.1186/s13321-023-00681-4

**Published:** 2023-02-02

**Authors:** Vladimir Kondratyev, Marian Dryzhakov, Timur Gimadiev, Dmitriy Slutskiy

**Affiliations:** 1 Computer Science and Artificial Intelligence Laboratory, ENGIE Lab CRIGEN, 4 rue Josephine Baker, 93240 Stains, France; 2 Telecom Paris, 19 Place Marguerite Perey, CS 20031, 91123 Palaiseau, France; 3 Laboratory of Chemoinformatics and Molecular Modeling, Butlerov Institute of Chemistry, Kazan Federal University, 18 Kremlyovskaya str., 420008 Kazan, Russia; 4 Federal Research Center “Kazan Scientific Center of Russian Academy of Sciences”, 420008 Kazan, Russia; 5 JSC “BIOCAD”, Petrodvortsoviy District, Strelna, Svyazi St., Bld. 34, Liter A., 198515 St. Petersburg, Russia

**Keywords:** GNN, JT-VAE, Structure optimization, HOMO energy, Molecular design

## Abstract

In this work, we provide further development of the junction tree variational autoencoder (JT VAE) architecture in terms of implementation and application of the internal feature space of the model. Pretraining of JT VAE on a large dataset and further optimization with a regression model led to a latent space that can solve several tasks simultaneously: prediction, generation, and optimization. We use the ZINC database as a source of molecules for the JT VAE pretraining and the QM9 dataset with its HOMO values to show the application case. We evaluate our model on multiple tasks such as property (value) prediction, generation of new molecules with predefined properties, and structure modification toward the property. Across these tasks, our model shows improvements in generation and optimization tasks while preserving the precision of state-of-the-art models.

## Introduction

Deep learning (DL) algorithms hold the promise of further accelerating advancements in almost every aspect of scientific research. Recent architectural developments in deep neural networks (DNN) [1] allowed for new applications beyond its initial targets in image recognition and text processing. As evident from the recent boost in the number of relevant publications [2], the field of chemistry has proved a fruitful ground for the application of the algorithms originally developed for natural language processing (NLP) [3] and graph processing (GP) [4] purposes. Chemical objects emerged as the natural extension of these algorithms due to the common representation of molecules as SMILES (text representation) [5] or molecular graphs (from valence theory). Both NLP and GP methodologies provide predictive and generative capabilities, thus paving the way for tackling the challenging problems of predicting molecular structures based on the desired chemical property.

In this study, we set on the task of developing a model for the predictive generation of molecular structures with the desired chemical property. As a property of interest, we have chosen to focus on the highest occupied molecular orbitals (HOMO) of small organic molecules due to the impact these electronic orbitals have on the physicochemical properties of molecules. The HOMO energy levels affect the reactivity and stability of chemical compounds, influence the properties of materials—for example, photovoltaic materials—and intimately impact the efficiency of light-to-electricity conversion in solar cells [6]. Our motivation is to facilitate the sampling of the virtual chemical space by developing an extended DL approach that encompasses the HOMO prediction task and moves the generation of new molecules from an explicit enumeration of all possible compounds to a refined small set with predefined HOMO properties.

Using Density Functional Theory (DFT) computational methods, it is possible to calculate HOMO energies of photovoltaic materials [7], yet these computations are costly in resources and time. In contrast, Machine Learning techniques allow fast and accurate prediction of HOMO levels. A comprehensive and detailed review of various machine learning techniques applied for the prediction of molecular orbital characteristics is given in [8]. In particular, Kernel Ridge Regression (KRR) is used in [9–15], Gaussian process regression (GPR), linear regressions (Elastic Net, Bayesian Ridge Regression) and Random Forest (RF) are applied in [14, 16, 17], respectively. Deep Learning techniques were used in [15, 18–26], including those based on graph molecular representations [21, 23]. The previously developed models set the regression task as the principal goal. To the best of our knowledge, no generation model for the molecules with the given HOMO level has been developed yet. Our technique is based on the junction tree variational autoencoder (JT VAE) [27] architecture which uses graph molecular representations as a reliable way of reproducing chemically valid structures. Our model achieves state-of-the-art results in HOMO energy prediction and allows the generation of new molecules with desired HOMO value. Several strategies for discovering chemical structures in the embedding space were suggested and explored.

## Model

### VAE and regression

**Fig. 1:**
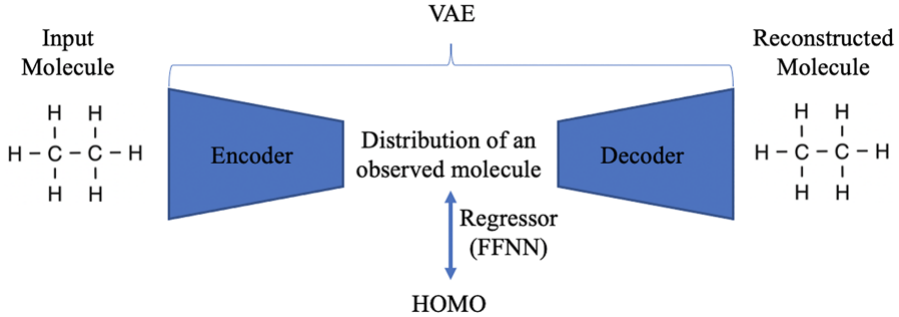
Simplified VAE architecture

Adapting from the work of [28], we use the variational auto-encoder neural network [29] to build our model. To this end, we train modified message passing networks to encode the molecular graph and a GRU-based message passing network to decode it. This approach produces mappings from the space of molecules to the embedding vector space and back, allowing us to later train the regression model from the embedding space and determine the molecular property of interest (see Fig. 1).

For the regression, we use a feedforward neural network (FFNN) with two hidden layers of size 1024.

### Junction tree VAE

In VAE training on graphs, the reconstruction of a molecule is a key challenge arising from the variability and complexity of molecular structures. The choice of architectures to efficiently reconstruct a graph is typically limited. In the present work, the graph is reconstructed in a sequence-to-sequence type model with a modified gated recurrent unit block [27]. One of the challenges of this approach lies in the possible formation of long sequences of nodes in the initial graph. These sequences are hard to encode and even harder to reconstruct accurately. For example, molecules with cyclic carbon structures proved challenging for accurate decoding because of the combinatorial variety of ring placements and side chain arrangements. Although it is still possible to apply graph encoding and decoding methods, they perform poorly. To simplify the molecular graph trees and eliminate all the complex molecular patterns, we utilize a junction tree mechanism that constructs an underlying tree-like reduced graph structure (hence the name) by an algorithm that maps the molecule into a unique tree representation and back. The nodes of the junction tree are chemical substructures with a rigid (fixed) spatial shape.

**Fig. 2:**
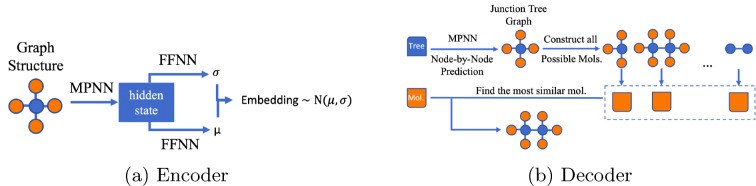
VAE components (FFNN—feed forward neural network, MPNN—message passing neural network)

**Fig. 3:**
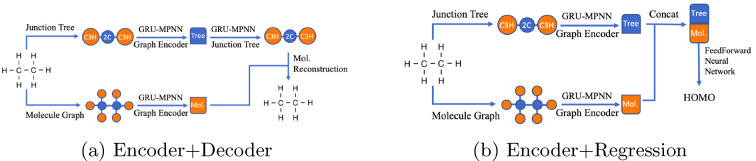
JT-VAE and regressor

In our approach, we use the encoded junction tree and the molecular graph to obtain independent embeddings for the molecule and its underlying structure (Fig. 2a). Thus, each molecule has two embeddings—one for the junction tree and one for the molecular graph which are concatenated to create one embedded representation of the molecule—a stacked vector used to perform regression and guided search for the suitable molecule in the embedded space (Fig. 3). The decoding procedure consists in creating a junction tree structure first and use the molecular graph hidden representation to guide the reconstruction of the junction tree into the final molecule (Fig. 2b).

### Architecture details

We present further details of our model in the following tables. Table 1 presents the configuration of model used to encode junction tree of a molecule. Table 2 presents the configuration for molecular encoder. Table 3 presents the configuration of regressor model. Table 4 presents the model used to decode the molecule from latent representation and model used to extract junction tree representation of reconstructed molecule for structures matching in decoding stage [27]. In the tables we write MPNN for modified Message Passing Neural Network [21, 27] and FFNN for Feed-Forward Neural Network.

**Table 1:** Junction tree encoder

Name	Input	Output	Info
One Hot Encoder	2327	612	–
MPNN	612	612	1 Iteration
FFNN	612	612	To extract features
FFNN	612	128	To extract mean
FFNN	612	128	To extract variance

**Table 2:** Molecular graph encoder

Name	Input	Output	Info
One Hot Encoder	50	612	–
MPNN	612	612	3 Iterations
FFNN	612	612	To extract features
FFNN	612	128	To extract mean
FFNN	612	128	To extract variance

**Table 3:** Regressor

Name	Input	Output
FFNN	612	1024
ReLU	–	–
FFNN	1024	1024
ReLU	–	–
BatchNorm1d	1024	1024
FFNN	1024	1

**Table 4:** Junction tree decoder

Name	Input	Output	Info
MPNN	256	612	–
FFNNN	612	1	For geometry prediction
FFNNN	612	2327	For structure prediction
MPNN	612	612	3 Iterations for molecule representation

## Training

The VAE model is designed to sample the latent embeddings from a probability distribution. The initial VAE training process of the encoder-decoder pair was conducted in two phases in analogy with [27]: first training the deterministic autoencoder network and later tuning the autoencoder model by adding a penalty term involving Kullback-Leibler divergence between latent vectors and standard normal distribution with a fixed penalty coefficient. As in the work of [27], our experiments indicate this approach as the optimal way of training.

A crucial factor for the training process was the training order of the encoder, decoder, and regressor triplet. We tried three strategies to ensure consensus between the regression and VAE model.

***Enc***, ***Dec*** $\rightarrow$ ***FFNN***

**Fig. 4:**
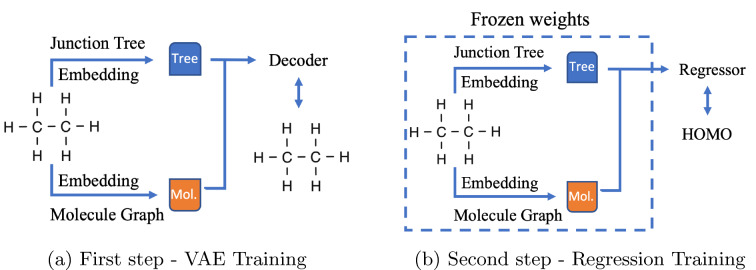
Finetuning of regression

The first strategy (Fig. 4) is simply when the feed forward neural network regression with ReLu activation (FFNN) is trained from the latent space to the property value space. Here the pretrained VAE encoder and decoder layers are set frozen.

**Fig. 5:**
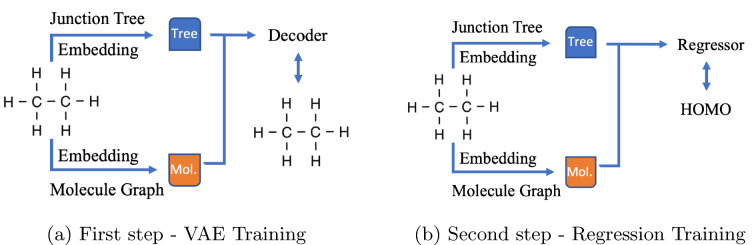
Training of regression

**Fig. 6:**
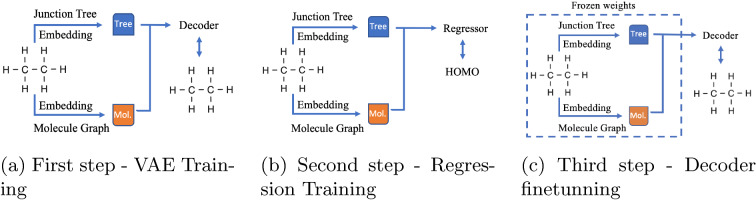
Training of decoder and regressor

***Enc***, ***Dec*** $\rightarrow$ ***Enc***, ***FFNN***

The second strategy (Fig. 5) was the VAE pair encoder-decoder training with a subsequent training of a VAE pair encoder-regressor; this way, we obtain a finetuned encoder, but the VAE’s decoder remains unchanged.

***Enc***, ***Dec*** $\rightarrow$ ***Enc***, ***FFNN*** $\rightarrow$ ***Dec***

The third strategy (Fig. 6) was a modified second one with an added step of retraining the encoder-decoder pair while keeping frozen the finetuned VAE encoder; we obtained our finetuned decoder in this way.

## Prediction of molecular structures with a given property value

The main problem addressed in this work is the accurate reconstruction of molecular structures from a given value of a chemical property of interest, namely HOMO energy values. There are two related issues in reverse quantitative structure-activity relationship (reverse QSAR), the first one is the existence of a molecule with the given property value, and the second one is the choice of the most interesting structure in the case when several molecules with the same HOMO value exist. We assume a molecule exists for each given HOMO value. In an ideal case, we would have to deal with a differentiable mapping *f* from the space *G* of molecules to the space *V* of its real-valued HOMO values. Assume that $v_0$ is the desired HOMO value. Then, for a molecule *g*, we can introduce a loss function $L_{v_0}(g) = |v_0 - f(g)|_p$ for some norm $|\cdot |_p$. In such a scenario, it would be possible to search for the optimal structure, by minimizing the $L_v$ using gradient descent methods. Unfortunately, since the space *G* of molecules is discrete, the mapping *f* is not differentiable, and we cannot apply the optimization approach directly. We use a VAE-type architecture to firstly map molecules from *G* to the corresponding latent space of the representations in $R_n$ for some *n* (encoder mapping $E: G \xrightarrow []{} R_n$) and back from $R_n$ to *G* (decoder mapping $D: R_n \xrightarrow []{} G$). Next, we construct regression mapping $f_R: R_n \xrightarrow []{} V$ and apply the above mentioned optimization process to the function $f_R$.

**Fig. 7:**
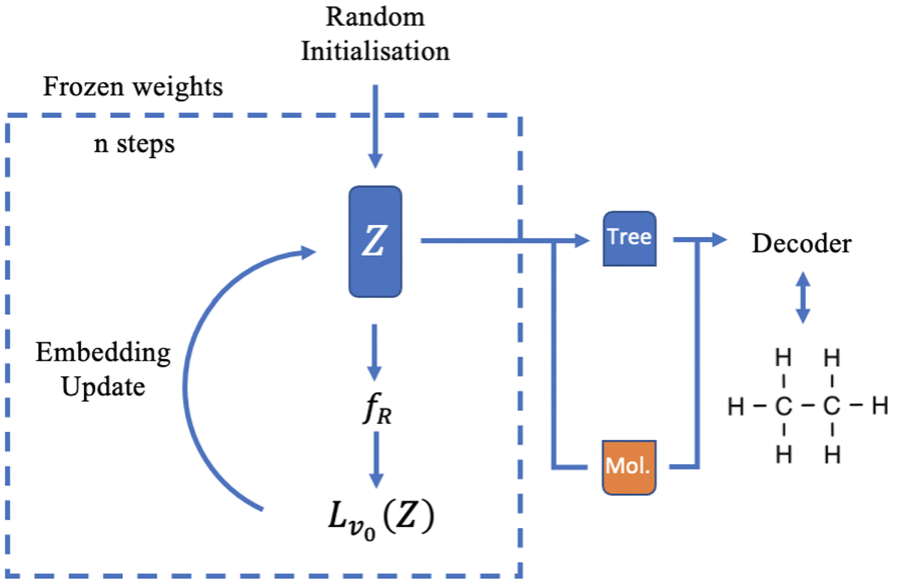
Latent space gradient descent

All these functions *E*, *D*,  and $f_R$ can be realized as neural networks. Once the models are trained (see Sect. "Training"), we can apply the auto differentiation techniques [28] to the neural network $f_R$ in order to do search in the molecule embedding space $R_n$. The minimization of $L_{v_0}$ will consist in fixing the weights of neural network and varying the argument *Z* in the direction of the gradient of $L_{v_0}(Z)$ in $R_n$. Once the gradient descent converged to some vector $Z_0$ in $R_n$ such that $f_R(Z_0)$ is close enough to $v_0$, we can reconstruct a molecule $D(Z_0)$ by applying the decoder mapping *D* (Fig. 7).

We have used three initialization approaches and two methods of structure optimization for the prediction of molecules from the test dataset. That resulted in six methods for searching molecules with prescribed property values.

### Vector initialization in the embedding space

We had to choose a method of initialization of the embedding vector that is to be fed to the gradient descent procedure described above. A good initialization of an embedding vector is of crucial importance to the result of the algorithm since the embedding space can have multiple minima—say optimal molecules—or no minimum for a given output value. Also, there is no intrinsic dependability that the local extreme must correspond to the molecule with the best fitting property. Fortunately, due to the inherent properties of VAE architecture, similar structures tend to locate close to each other in the embedding space.

In our work, we studied different approaches to initialize a molecule (and thus the corresponding embedding vector). We introduce the following notion: let $\text {REGR}_{\text {TR}}$ and $\text {REGR}_{\text {VAL}}$ be datasets used in the regression training procedure, CTV—a molecule from $\text {REGR}_{\text {TR}}$ closest by target property value, CHV—a molecule from $\text {REGR}_{\text {TR}}$ closest by hidden vector in the latent space to a given one.

**Fig. 8:**
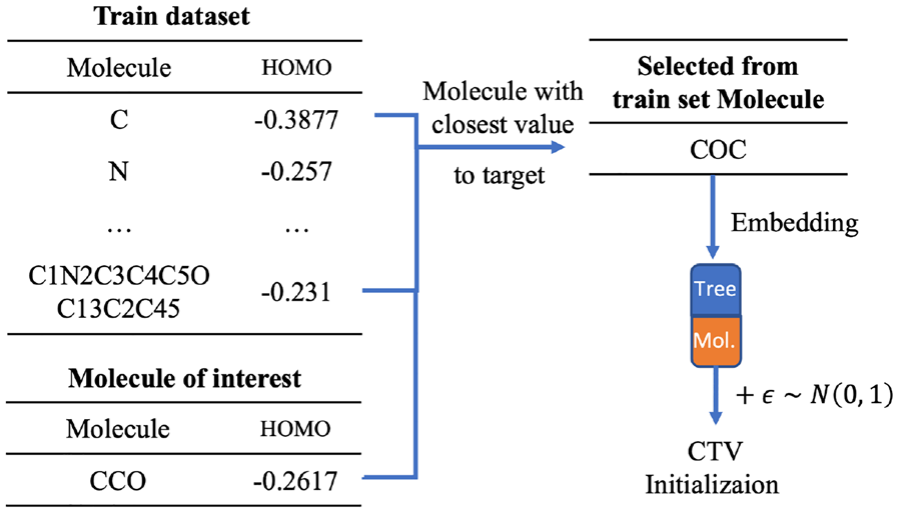
Closest by value initialization

**Fig. 9:**
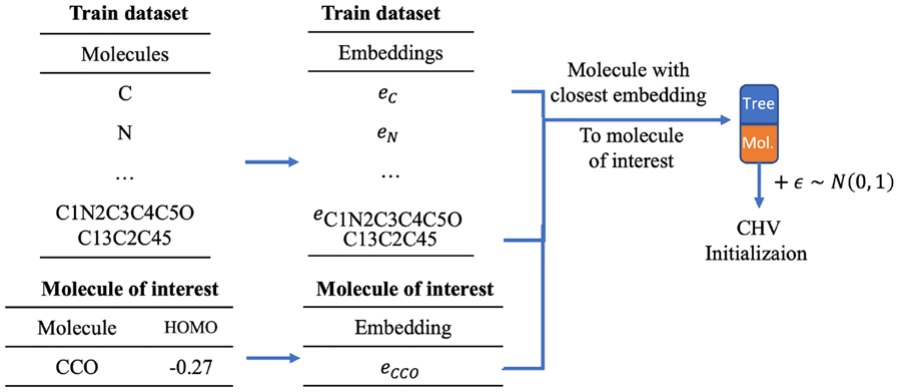
Closest by hidden vector initialization

***Gaussian***: The initial approach consisted in sampling the vector in the latent space $R_n$ from a normal multinomial distribution with a 1-diagonal covariance matrix.
***CTV + Gaussian***: We assume that the molecules with similar properties should have close projections in the embedding space $R_n$. Thus, if we wish to invent molecules with the property value $v_0$, then we search molecules in the train set $\text {REGR}_{\text {TR}}$ with the property values the closest to $v_0$, and we use their embeddings in $R_n$. We applied the centered Gaussian noise with 1-diagonal covariance matrix to these starting vectors in order to augment the set of initializations in $R_n$ (Fig. 8).
***CHV + Gaussian***: This initialization method is good to simulate the generation of a molecule with a given property value which belongs to some particular class of chemical compounds. We take some molecule $m_{int}$ of a given class, and we start the gradient descent with a molecule from $\text {REGR}_{\text {TR}}$ which has the closest $L_2$ distance to the embedding of $m_{int}$. In our experiments we used the extreme case of this initialization method trying to reconstruct molecules from the validation set (Fig. 9).

### Molecule optimization algorithms

Now we describe the optimization algorithms we used.

Algorithm A is a formalization of the gradient descent in the molecule embedding space described in the beginning of Sect. "Prediction of molecular structures with a given property value".
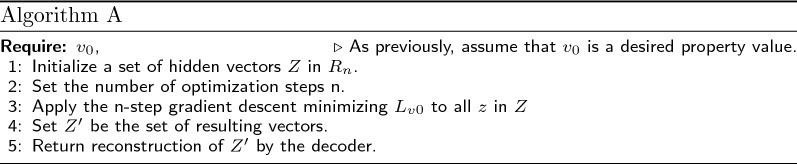


According to the VAE paradigm, any molecule corresponds not just to one vector, but to a domain in the embedding space $R_n$. However, the regression function attributes different property values to each embedding, thus making the prediction ambiguous. We used the mean vector of normal distribution created by the VAE architecture as an input for the regression model. After the convergence in the latent space, the resulting vector does not a priori correspond to the mean of any molecule. A natural way to go around this problem is to decode the molecule from the found vector and then apply the encoder to calculate the mean embedding vector of the predicted molecule. Following this intuition, we suggest Algorithm B for molecule optimization. This approach allows us to look for an embedding that corresponds to a mean of some molecule and has the closest possible property value to the value of interest.
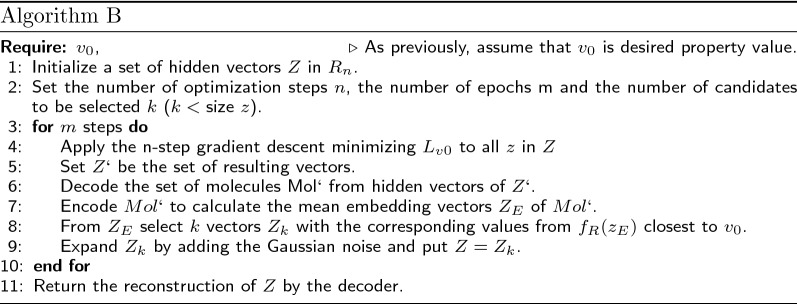


## Datasets

We use the ZINC [30] and QM9 [31] datasets in our work.

The QM9 dataset [31] is a widely used benchmark for the prediction of physical properties of molecules in equilibrium state. It consists of around 130k small organic molecules with up to nine heavy atoms of C, O, N, or F with the properties computed using DFT calculations. The QM9 contains additional information, from which we also obtain the HOMO properties of molecules to perform the regression task and test our structure optimization approach.

The ZINC dataset [30] is a subset of a free database [32] of commercially available compounds for virtual screening.

*A dataset mixture to train the encoder-decoder pair.* Originally in [27], JT VAE was studied by utilizing the ZINC dataset [30]. It was discovered that ZINC lacks a variety of molecule types to the extent that the models trained on it lack efficiency in real tasks [8]. To avoid the influence of a bias of a particular dataset on the training, we have extended the previous approach to a mixture of QM9 and ZINC datasets (we call it MIX) which helped us improve the model’s generalization properties and general accuracy of reconstruction.

JT VAE method decomposes every molecule into building blocks. One of the central assumptions that we used in our work is that the dictionary of simple structures forming the molecules is large enough for decomposing any possible molecule. This assumption is rarely satisfied when we change from one chemical database to another. Therefore, for the MIX dataset we expanded the vocabulary of simple compounds as much as possible. In particular, we grow the dictionary from 780 original building blocks covering the ZINC database in JT VAE to 2327 basic objects spanning MIX. The total number of molecules in MIX is 355796.

*Datasets used to train the regression.* For the property of interest - e.g., molecular HOMO energy levels - merging two different databases is often a challenging task since there is no unique way to correctly join the data from various sources due to variation in theoretical calculation techniques, conditions of measurements, etc. To overcome the consistency issues, we performed the regression training on QM9 dataset only.

First, we have partitioned the $\text {QM9}$ datasets on the train, validation and test subsets ($\text {QM9}_{\text {TR}}$, $\text {QM9}_{\text {VAL}}$, and $\text {QM9}_{\text {TEST}}$, respectively), so that the validation part contains 2500 molecules, and the test part contains 5000 molecules. For the consistency reasons, VAE was trained on the combined $\text {ZINC} + \text {QM9}_{\text {TR}}$ dataset, and $\text {QM9}_{\text {VAL}}$ was used for validation; for the training procedure of regression, $\text {QM9}_{\text {TR}}$ and $\text {QM9}_{\text {VAL}}$ were used; $\text {QM9}_{\text {TEST}}$ was used as test data across HOMO prediction and reconstruction of molecules.

## Results

### Basic auto encoding

In Table 5 we give the results of a basic JT-VAE training. The columns *Train* and *Test* correspond to the respective datasets used in the process. The column *Acc* contains the percentage of accurately reconstructed molecules by the encoder decoder pair. The column *Chemical Validity* represents the chemical validity of obtained molecules. The column *KL* indicates whether the Kullback-Leibler divergence penalty term was used in the second stage of the training (see Subsection * VAE and Regression*).

**Table 5:** Results of reconstruction accuracy

Train	Test	Acc (%)	Chemical validity (%)	KL
MIX	QM9	83.1	100	True
QM9	QM9	81.9	100	True
QM9	QM9	79.4	100	False
MIX	QM9	81	100	False
ZINC	ZINC	75	100	True

The results of the basic encoder-decoder training presented in Table 5 serve as a baseline for a multi-step VAE training described in Sect. "Training".

### Regression and multi-step auto encoding

Table 6 represents results mentioned in [8] for different datasets and methods of HOMO energies prediction, which were trained and tested on the indicated datasets. More complete description of the methods can be found in [8]. The last four lines colored in gray correspond to our results. The lines *Ridge Regr.* and *Elastic Net* correspond to the training of the eponymous regression from latent space into the property of interest (HOMO). The last two lines correspond to the training of the unfrozen encoder (pretrained during the basic JT VAE training) jointly with two layers feed forward neural network with ReLu activations.

We measure the HOMO accuracy (*HOMO Acc* columns in Tables 6 and 7) by *MAE* loss and the VAE accuracy (*VAE Acc* column in Table 7) by finding the percentage of SMILES strings that represent the molecule after reconstruction that are the same as initial SMILES strings.

**Table 6:** Results of regression accuracy

Study	Method	Train	Test	HOMO Acc
Mentioned in [8]	Elastic Net	PC9	PC9	0.47
	Ridge Regr.	PC9	PC9	0.31
	SchNet	PC9	PC9	0.06
	SchNet	QM9	PC9	0.07
	SchNet	QM9	PC9	0.33
	SchNet	PC9	QM9	0.05
	SchNet	PC9	QM9	0.12
	SchNet	PC9	QM9	0.12
	SchNet	QM9	PC9	0.3
	SchNet	QM9	QM9	0.04
This study	Ridge Regr.	QM9	QM9	0.18
	Elastic Net	QM9	QM9	0.34
	JT-ENC + FFNN	QM9	QM9	0.09
	JT-ENC + FFNN	MIX	QM9	0.09

In Table 7 we give the results for the joint VAE + regressor training. Three strategies were introduced in Section *Training*. We figure out that the third strategy is a good trade-off for the quality of the regressor’s property value prediction and the molecule reconstruction accuracy by the encoder-decoder pair. Note that $0\%$ VAE Accuracy in the second line of Table 7 is due to the fact that the encoder trained together with the regressor does not match the decoder anymore, therefore the third joint VAE and Regression training strategy was introduced. The best regression [22] from Table 6 performs slightly better than our JT-ENC+FFNN regression model, but coupled with the decoder, our model can generate new molecules which is not possible for other researchers’ models in Table 6.

**Table 7:** Comparison of joint VAE and Regression training strategies

Order	Train	Test	HOMO Acc	VAE Acc (%)
Enc, Dec$\xrightarrow []{}$FFNN	MIX	QM9	0.32	84
Enc, Dec$\xrightarrow []{}$Enc, FFNN	MIX	QM9	0.09	0
Enc, Dec$\xrightarrow []{}$Enc, FFNN$\xrightarrow []{}$Dec	MIX	QM9	0.09	81

### Molecule reconstruction

For Table 8 the methods of the columns *Initialization* and *Type of search* are described in Subsection *Vector initialization in the embedding space*, and the column *Reconstruction* displays the rate of molecules predicted from $\text {QM9}_{\text {TEST}}$ dataset.

**Table 8:** Experiments on type of initialization

Initialization	Type of search	Reconstruction (%)
Gaussian	A	0.04
CTV + Gaussian	A	0.04
CHV + Gaussian	A	2
Gaussian	B	0
CTV + Gaussian	B	0
CHV + Gaussian	B	0.08

**Fig. 10:**
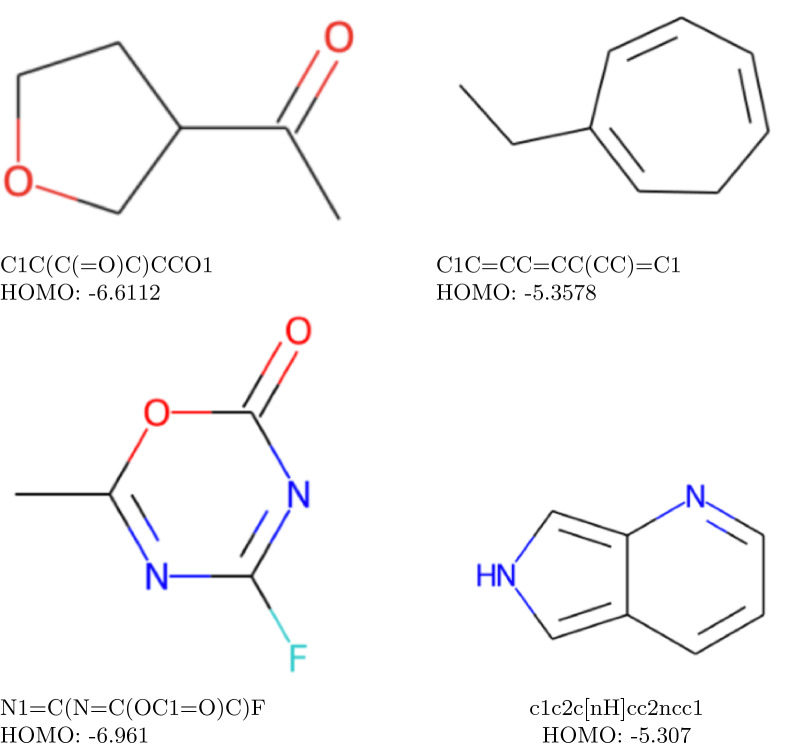
Reconstructed molecules from $\text {QM}9_{\text {val}}$ dataset. The target HOMO value is the same as the predicted HOMO value, that is specified under molecules

The low values of the reconstruction percentage column in Table 8 are due to the fact that the model performed the unguided structure search for the HOMO values of the molecules from the $\text {QM9}_{\text {TEST}}$ dataset, and also since multiple molecules can have close (or equal) HOMO values. We are unable to compute the real HOMO values of all the molecules suggested by our model because of high costs of DFT computations, but we attest that our model generated correctly the “reconstructed” molecules according to their HOMO values. Given that the $\text {QM9}_{\text {TEST}}$ dataset was never exposed to our model during the training, we argue that the implementation of our algorithm warrants both the validity of predicted molecules and the high accuracy of HOMO value prediction. In Fig. 10 we give several molecules from the $\text {QM9}_\text {TEST}$ dataset that were suggested by our algorithm and thus we know that their HOMO values were predicted perfectly.

**Fig. 11:**
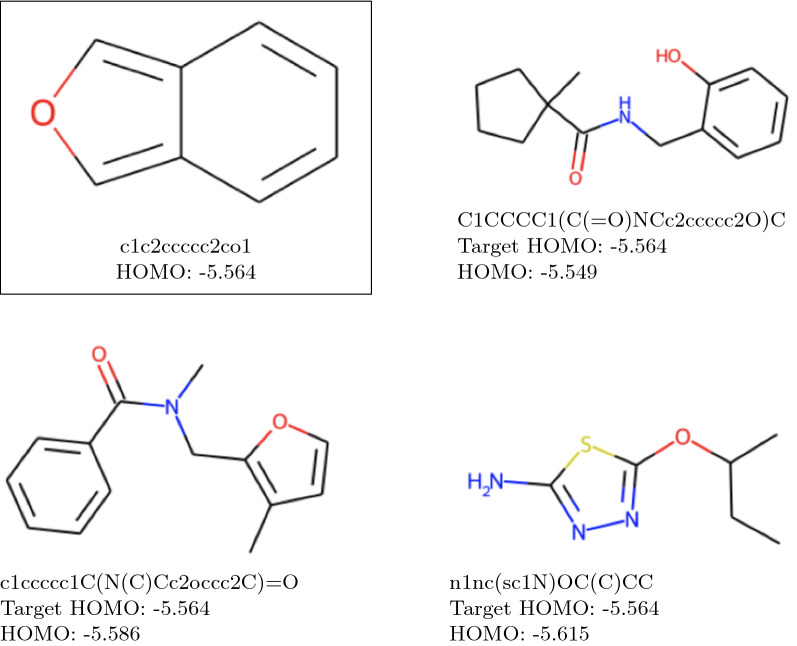
Reconstruction of a molecule from an independent data set and alternative structures

In addition, we tested method B with a combination of CHV and Gaussian initialization on independent data. To this purpose, we took a molecule from [33] that is not in any way connected to MIX or QM9 datasets. The proposed method has successfully reconstructed the selected molecule c1c2ccccc2co1 from [33], which corroborates the reliability of our method. In Fig. 11 we give the reconstructed molecule and other examples of proposed structures. The molecule of interest is surrounded with a square.

**Fig. 12:**
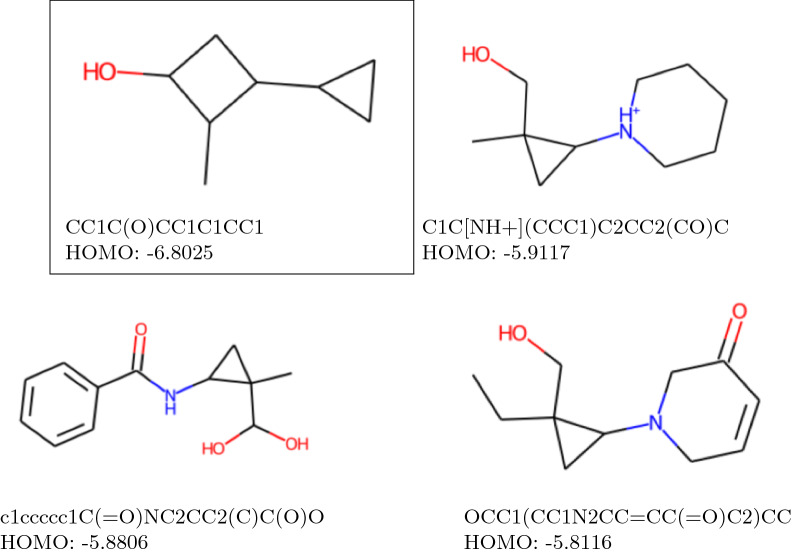
Guided optimization

### Guided structure optimization

Guided structure optimization is a particular case of CHV; the concept is that we modify some molecules to obtain similar molecules with desired properties. With JT VAE architecture, we can influence how the molecular structure changes since the molecular decomposition is fully determined by a hidden vector that represents the junction tree. By modifying this vector slightly, we can introduce a change in the molecule, thus enhancing a certain property value. For instance, in Fig. 12 we showcase the molecules (together with their HOMO values predicted by our regressor model) that we obtained after 100 iterations of the algorithm B applied to the arbitrarily chosen molecule CC1C(O)CC1C1CC1 from MIX, with the base HOMO value of -6.8025, when the target value was arbitrarily set to be -5.93722 (the original molecule is marked with a square on the image).

### Conclusion

The proposed model allows the prediction of important chemical property values, namely the HOMO energy levels, which are otherwise costly to compute. While our model performs on the same level of accuracy as the current state-of-the-art regression models, the junction tree variational autoencoder coupled with a regression algorithm also allows to create new molecular structures with desired HOMO value. We have proposed two strategies for performing the guided search for such structures and experimentally shown that our model produces molecules with desirable target properties.

### Further developments

In our work we explore the applications of the JT-VAE architecture [27] for molecule design. It allows expansions in various directions, like choosing different functions that perform gradient descent and also multi-criteria search (for instance, for HOMO and LUMO energies simultaneously), diffetent graph VAE architectures, or, instead, Generative Adversarial Networks (GANs) [34, 35], and Wasserstein GANs (WGANs) [36, 37].

## Data Availability

The code was written in Python 3.8, using RDKit library and it was based on the Junction Tree Variational Autoencoder implementation from [27]. Our code together with the utilized datasets are available at https://github.com/VldKnd/vae_qm9.

## References

[1] Dong S, Wang P, Abbas K (2021). A survey on deep learning and its applications. Comput Sci Rev.

[2] Karthikeyan A, Priyakumar U (2022). Artificial intelligence: machine learning for chemical sciences. J Chem Sci.

[3] Hedderich MA, Lange L, Adel H, Strötgen J, Klakow D (2021) A survey on recent approaches for natural language processing in low-resource scenarios. In: Proceedings of the 2021 Conference of the North American Chapter of the Association for Computational Linguistics: Human Language Technologies, pp. 2545– 2568. Association for Computational Linguistics, Online. 10.18653/v1/2021.naacl-main.201. https://aclanthology.org/2021.naacl-main.201https://aclanthology.org/2021.naacl-main.201

[4] Wu Z, Pan S, Chen F, Long G, Zhang C, Yu PS (2021). A comprehensive survey on graph neural networks. IEEE Trans Neural Netw Learn Syst.

[5] Weininger D (1998). Smiles, a chemical language and information system. 1. Introduction to methodology and encoding rules. J Chem Inf Comput Sci.

[6] Jo MY, Park SJ, Park T, Won YS, Kim JH (2012). Relationship between homo energy level and open circuit voltage of polymer solar cells. Org Electron.

[7] Setsoafia DDY, Ram KS, Mehdizadeh-Rad H, Ompong D, Murthy V, Singhs J (2022). Dft and td-dft calculations of orbital energies and photovoltaic properties of small molecule donor and acceptor materials used in organic solar cells. J Renew Mater.

[8] Glavatskikh M, Leguy J, Hunault G, Cauchy T, Da Mota B (2019). Dataset’s chemical diversity limits the generalizability of machine learning predictions. J Cheminform.

[9] Rupp M, Tkatchenko A, Müller K-R, von Lilienfeld OA (2012). Fast and accurate modeling of molecular atomization energies with machine learning. Phys Rev Lett.

[10] Hansen K, Montavon G, Biegler F, Fazli S, Rupp M, Scheffler M, von Lilienfeld OA, Tkatchenko A, Müller K-R (2013). Assessment and validation of machine learning methods for predicting molecular atomization energies. J Chem Theory Comput.

[11] Hansen K, Biegler F, Ramakrishnan R, Pronobis W, von Lilienfeld OA, Müller K-R, Tkatchenko A (2015). Machine learning predictions of molecular properties: accurate many-body potentials and nonlocality in chemical space. J Phys Chem Lett.

[12] Ramakrishnan R (2015) v.L.O.: Many molecular properties from one kernel in chemical space. Chimia (Aarau) 10.2533/chimia.2015.182 26672132

[13] Huang B, von Lilienfeld OA (2016). Communication: Understanding molecular representations in machine learning: the role of uniqueness and target similarity. J Chem Phys.

[14] Faber FA, Hutchison L, Huang B, Gilmer J, Schoenholz SS, Dahl GE, Vinyals O, Kearnes S, Riley PF, von Lilienfeld OA (2017). Prediction errors of molecular machine learning models lower than hybrid dft error. J Chem Theory Comput.

[15] Collins CR, Loyd Gordon GJ (2018). Constant size descriptors for accurate machine learning models of molecular properties. J Chem Phys.

[16] Bartók AP, De S, Poelking C, Bernstein N, Kermode JR, Csányi G, Ceriotti M (2017). Machine learning unifies the modeling of materials and molecules. Sci Adv.

[17] Montavon G, Rupp M, Gobre V, Vazquez-Mayagoitia A, Hansen K, Tkatchenko A, Müller K-R, von Lilienfeld OA (2013). Machine learning of molecular electronic properties in chemical compound space. New J Phys.

[18] Unke OT, Meuwly M (2019). Physnet: a neural network for predicting energies, forces, dipole moments, and partial charges. J Chem Theory Comput.

[19] Smith JS, Isayev O, Roitberg AE (2017). Ani-1: an extensible neural network potential with dft accuracy at force field computational cost. Chem Sci.

[20] Pereira F, Xiao K, Latino DARS, Wu C, Zhang Q, Aires-de-Sousa J (2017). Machine learning methods to predict density functional theory b3lyp energies of homo and lumo orbitals. J Chem Inf Model.

[21] Gilmer J, Schoenholz SS, Riley PF, Vinyals O, Dahl GE (2017) Neural message passing for quantum chemistry. In: Proceedings of the 34th International Conference on Machine Learning, Vol 70, pp. 1263– 1272. JMLR.org

[22] Schütt KT, Kindermans P-J, Sauceda HE, Chmiela S, Tkatchenko A, Müller K-R (2017) Schnet: a continuous-filter convolutional neural network for modeling quantum interactions. Adv Neural Inf Process Syst 30:992–1002. 10.48550/ARXIV.1706.08566

[23] Hy TS, Trivedi S, Pan H, Anderson BM, Kondor R (2018). Predicting molecular properties with covariant compositional networks. J Chem Phys.

[24] Hy TS, Trivedi S, Pan H, Anderson BM, Kondor R, Hou F, Wu Z, Hu Z, Xiao Z, Wang l, Zhang X, Li G (2018) comparison study on the prediction of multiple molecular properties by various neural networks. J Chem Phys. 10.1021/acs.jpca.8b09376 10.1021/acs.jpca.8b09376 30285444

[25] Lubbers N, Smith JS, Barros K (2018). Hierarchical modeling of molecular energies using a deep neural network. J Chem Phys.

[26] Unke OT, Meuwly M (2018). A reactive, scalable, and transferable model for molecular energies from a neural network approach based on local information. J Chem Phys.

[27] Jin W, Barzilay R, Jaakkola T (2019) Junction tree variational autoencoder for molecular graph generation 1802:04364

[28] Gómez-Bombarelli R, Wei JN, Duvenaud D, Hernández-Lobato JM, Sánchez-Lengeling B, Sheberla D, Aguilera-Iparraguirre J, Hirzel TD, Adams RP, Aspuru-Guzik A (2018). Automatic chemical design using a data-driven continuous representation of molecules. ACS Cent Sci.

[29] Kingma DP, Welling M (2014) Auto-encoding variational bayes. In: 2nd International Conference on Learning Representations, ICLR 2014, Banff, AB, Canada, April 14–16, 2014, Conference Track Proceedings. arXiv:http://arxiv.org/abs/1312.6114v10

[30] Kusner MJ, Paige B, Hernández-Lobato JM (2017) Grammar variational autoencoder. In: Proceedings of the 34th International Conference on Machine Learning—Volume 70. ICML’17, pp. 1945– 1954. JMLR.org

[31] Ramakrishnan R, Dral PO, Rupp M, von Lilienfeld OA (2014) Quantum chemistry structures and properties of 134 kilo molecules. Sci Data 1 10.1038/sdata.2014.22 PMC4322582 25977779

[32] Irwin JJ, Sterling T, Mysinger MM, Bolstad ES, Coleman RG (2012). Zinc: a free tool to discover chemistry for biology. J Chem Inf Model.

[33] Margetic D DPW, Warrener RN (2004). Diels-alder reactivity of benzannulated isobenzofurans as assessed by density functional theory. J Mol Model.

[34] De Cao N, Kipf T (2018) MolGAN: an implicit generative model for small molecular graphs. arXiv: 1805.11973

[35] Łukasz Maziarka Pocha A, Kaczmarczyk J, Warchoł M(2019) Mol-CycleGAN—a generative model for molecular optimization (2019). https://openreview.net/forum?id=BklKFo09YX 10.1186/s13321-019-0404-1 PMC6950853 33431006

[36] Arjovsky M, Chintala S, Bottou L (2017) Wasserstein generative adversarial networks. In: Precup D, Teh YW (eds) Proceedings of the 34th International conference on machine learning. Proceedings of Machine Learning Research, vol. 70, pp. 214–223. PMLR. https://proceedings.mlr.press/v70/arjovsky17a.html

[37] Gulrajani I, Ahmed F, Arjovsky M, Dumoulin V, Courville AC ( 2017) Improved training of wasserstein gans. In: Guyon I, Luxburg UV, Bengio S, Wallach H, Fergus R, Vishwanathan S, Garnett R (eds.) Advances in Neural Information Processing Systems, vol. 30. Curran Associates, Inc. https://proceedings.neurips.cc/paper/2017/file/892c3b1c6dccd52936e27cbd0ff683d6-Paper.pdf

